# Comparing different machine learning techniques for predicting COVID-19 severity

**DOI:** 10.1186/s40249-022-00946-4

**Published:** 2022-02-17

**Authors:** Yibai Xiong, Yan Ma, Lianguo Ruan, Dan Li, Cheng Lu, Luqi Huang, the National Traditional Chinese Medicine Medical Team

**Affiliations:** 1 Institute of Basic Research in Clinical Medicine, China Academy of Chinese Medical Sciences, No. 16, Nanxiao Street, Dongzhimen, Dongcheng District, Beijing, 100700 Beijing China; 2 Department of Infectious Diseases, JinYinTan Hospital, Wuhan, 430040 China; 3 Information Center, Chinese Center for Disease Control and Prevention, Beijing, 102206 China; 4 National Resource Center for Chinese Materia Medica, China Academy of Chinese Medical Sciences, No. 16, Nanxiao Street, Dongzhimen, Dongcheng District, Beijing, 100700 Beijing China

**Keywords:** COVID-19, Severity, Machine learning, Support vector machine, Random Forest, Logistic regression

## Abstract

**Background:**

Coronavirus disease 2019 (COVID-19) is still ongoing spreading globally, machine learning techniques were used in disease diagnosis and to predict treatment outcomes, which showed favorable performance. The present study aims to predict COVID-19 severity at admission by different machine learning techniques including random forest (RF), support vector machine (SVM), and logistic regression (LR). Feature importance to COVID-19 severity were further identified.

**Methods:**

A retrospective design was adopted in the JinYinTan Hospital from January 26 to March 28, 2020, eighty-six demographic, clinical, and laboratory features were selected with LassoCV method, Spearman’s rank correlation, experts’ opinions, and literature evaluation. RF, SVM, and LR were performed to predict severe COVID-19, the performance of the models was compared by the area under curve (AUC). Additionally, feature importance to COVID-19 severity were analyzed by the best performance model.

**Results:**

A total of 287 patients were enrolled with 36.6% severe cases and 63.4% non-severe cases. The median age was 60.0 years (interquartile range: 49.0–68.0 years). Three models were established using 23 features including 1 clinical, 1 chest computed tomography (CT) and 21 laboratory features. Among three models, RF yielded better overall performance with the highest AUC of 0.970 than SVM of 0.948 and LR of 0.928, RF also achieved a favorable sensitivity of 96.7%, specificity of 69.5%, and accuracy of 84.5%. SVM had sensitivity of 93.9%, specificity of 79.0%, and accuracy of 88.5%. LR also achieved a favorable sensitivity of 92.3%, specificity of 72.3%, and accuracy of 85.2%. Additionally, chest-CT had highest importance to illness severity, and the following features were neutrophil to lymphocyte ratio, lactate dehydrogenase, and D-dimer, respectively.

**Conclusions:**

Our results indicated that RF could be a useful predictive tool to identify patients with severe COVID-19, which may facilitate effective care and further optimize resources.

**Graphical Abstract:**

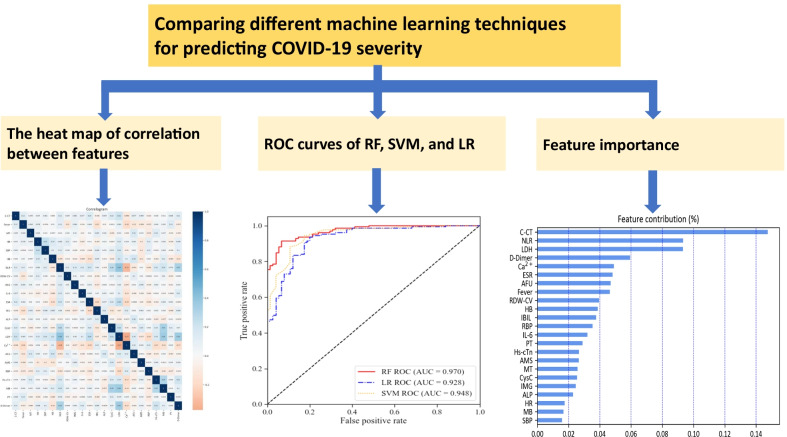

**Supplementary Information:**

The online version contains supplementary material available at 10.1186/s40249-022-00946-4.

## Background

Coronavirus disease 2019 (COVID-19) has been a severe public health event and is still ongoing spreading globally [1, 2]. The main manifestations of COVID-19 include fever, fatigue, and dry cough, most COVID-19 cases are non-severe with favorable outcome [3, 4]. However, patients with severe COVID-19 tend to progress rapidly and experience respiratory failure, respiratory distress syndrome, and septic shock or even death within a short period of time [5], with a high mortality of 53% [6]. Previous studies observed that male, aged over 65, smoking, elevated lactate dehydrogenase, elevated D-dimer, and chest imaging findings associated with COVID-19 severity [7–10]. However, the studies didn’t demonstrate indicators’ contribution to COVID-19 severity on admission. Hence, it is essential to assess importance of features to disease severity on admission for earlier and more targeted care, further reduce severe disease and prioritize medical resource.

Machine learning as an effective and innovative tool has been reported applications in diabetes [11], cardiovascular diseases [12], cancer [13, 14], sepsis [15], and depression [16], etc., and showed favorable performance. Moreover, machine learning was used to predict COVID-19 diagnosis and treatment outcome, etc. [17–21]. However, different machine learning techniques were used to predict COVID-19 severity have not well been reported. Our study aimed to compare different machine learning techniques including random forest (RF), support vector machine (SVM), and logistic regression (LR) to predict COVID-19 severity based on clinical, laboratory, and chest computed tomography (C-CT) features, further identify feature importance to COVID-19 severity. The results can provide reference for clinicians to diagnose and treat patients timely and effectively, and further optimize medical resources, especially in resource-limited areas.

## Methods

### Study design and participants

A retrospective study was conducted in the JinYinTan Hospital from January 26 to March 28, 2020. Data of demographic, clinical, laboratory, C-CT features, and treatment outcome were reviewed from 287 patients of hospital information system, all cases were laboratory-confirmed by real-time reverse transcriptase polymerase chain reaction assay.

### Feature selection

**Fig. 1:**
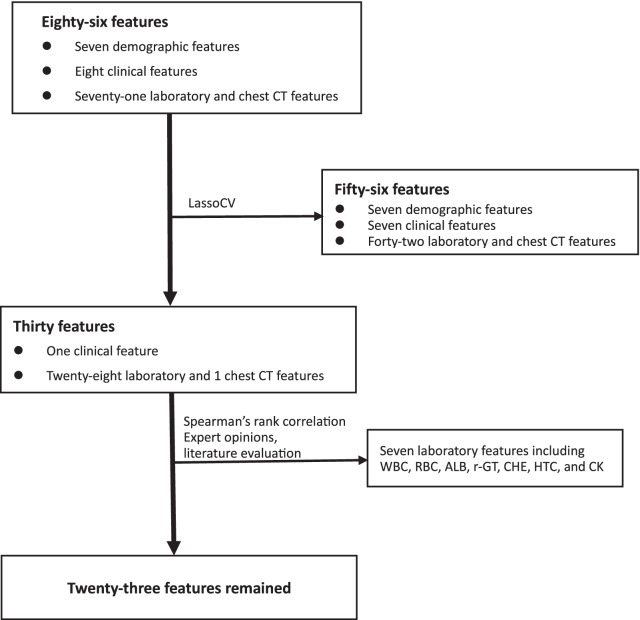
Flow chart of feature selection. *WBC* White blood cell, *RBC* red blood cell, *ALB* albumin, *r-GT* r-glutamyl transpeptidase, *CHE* cholinesterase, *HCT* hematocrit, *CK creatine* kinase

**Fig. 2:**
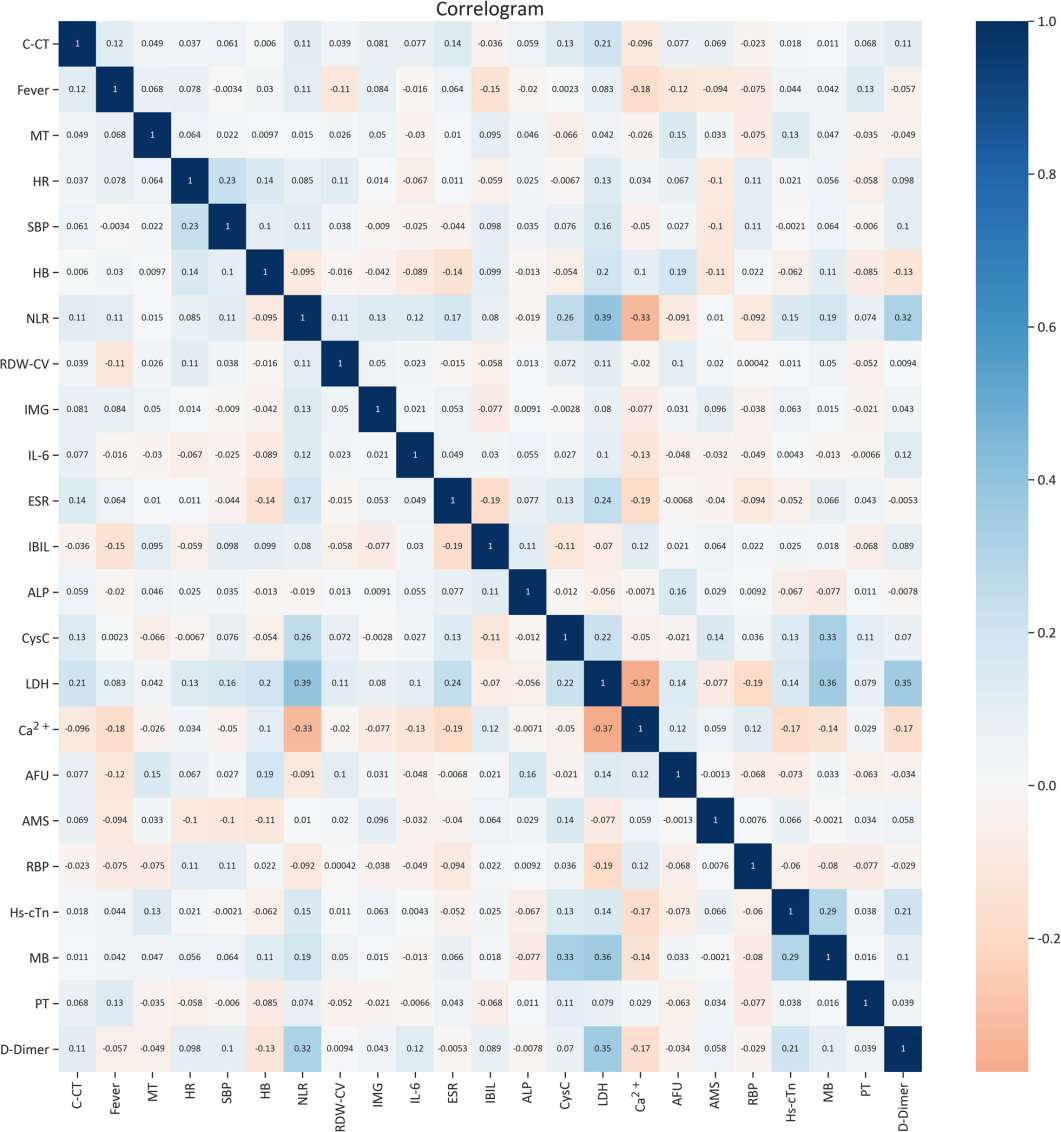
The heat map of correlation between features. Color indicates the value of the correlation coefficient (r). The color intensity is proportional to the correlation coefficient (r), with positive correlations (r > 0) shown and negative correlations (r < 0), lower intensive color indicates lower correlations, in the study, 23 features selected by machine learning techniques, each feature is weakly correlated with each other (r < 0.4). Twenty-three features including C-CT, fever, MT, HR, SBP, HB, NLR, RDW-CV, IMG, IL-6, ESR, IBIL, ALP CysC, LDH, Ca^2+^, AFU, AMS, RBP, Hs-cTn, MB, PT(s), D-dimer. *C-CT* chest computed tomography, *MT* malignant tumor, *HR* heart rate, *SBP* systolic blood pressure, *NLR* neutrophil-to-lymphocyte ratio, *HB* hemoglobin concentration, *RDW-CV* red cell volume distribution width, *LDH* lactate dehydrogenase, *IBIL* indirect bilirubin, *PT* prothrombin time, *ESR* erythrocyte sedimentation rate, *AFU* α-fucosidase, *RBP* retinol-Binding protein, *IL-6* interleukin-6, *Hs-cTn* hypersensitive troponin, *AMS* amylase, *CysC* cystatin C, *IMG* immature granulocyte, *ALP* alkaline phosphatas, *MB* myoglobin

In the present study, 30 features were remained after the initial 86 features were screened by LassoCV in the study. Subsequently, we carried out spearman’s rank correlation to further reduce confounding, features with correlation coefficients greater than 0.4 (r > 0.4)were excluded. For example, white blood cell (WBC) was correlated with neutrophil to lymphocyte ratio (NLR); red blood cell (RBC) and hematocrit (HCT) were both correlated with hemoglobin concentration (HB); albumin (ALB) is associated with cholinesterase (CHE), etc. Additionally, combined with expert opinions, and literature evaluation 7 features were excluded from 30 features, namely WBC, RBC, HCT, ALB, r-glutamyl transpeptidase(r-GT), CHE, creatine kinase (CK), then 23 features were remained (Fig. 1), correlation coefficient between features were less than 0.4 (Fig.2), which were entered into models for further analysis.

Twenty-three features included chest computed tomography (C-CT), fever, malignant tumor(MT), heart rate (HR), systolic blood pressure (SBP), hemoglobin concentration (HB), neutrophil-to-lymphocyte ratio (NLR), red cell volume distribution width (RDW-CV), immature granulocyte (IMG), interleukin-6 (IL-6), erythrocyte sedimentation rate (ESR), indirect bilirubin (IBIL), alkaline phosphatas (ALP), cystatin C (CysC), lactate dehydrogenase (LDH), Ca^2+^, α-fucosidase (AFU), amylase (AMS), retinol-Binding protein (RBP), hypersensitive troponin (Hs-cTn), myoglobin (MB), prothrombin time (PT), D-dimer.

### Features and models

Both dichotomous features are containing precisely two distinct values in the form of 0 and 1, for the three classification features such as C-CT, 0, 1 and 2 as three categorical variables were used to represent no lesions, unilateral lesions, and bilateral lesions, respectively; continuous variables were expressed with unit standardization. The 23 features were processed by RF, SVM, and LR modeling to compare the three models in predicting COVID-19 severity on admission.

RF is a classifier that contains multiple decision trees, and the output is determined by the voting results of multiple trees. We used a grid search method to determine the number of classifiers, maximum depth, and minimum number of branch samples in random forest modeling. SVM based on statistical learning theory, is a class of generalized linear classification technique that classifies data binary in a supervised learning manner, and correctly classifies samples by looking for decision boundaries. LR is a generalized linear regression analysis model that uses different kernel functions to estimate data conditions. Similar to random forest, we use grid search method to determine the selection of key parameters of SVM and LR, thereby reducing the training error in modeling.

### COVID-19 severity for prediction

COVID-19 severity was determined based on *the Diagnosis and Treatment Protocol for COVID-19 (seventh edition) *[22]. In our study, given the small sample of 287 patients analyzed from single center, therefore, COVID-19 cases were categorized as non-severe and severe cases. Mild and moderate cases were classified as non-severe cases, severe and critical were classified as severe cases. The outcome variable was coded as binary 0 or 1, and the predictive output variable was defined as severe type (coded as 1) and non-severe type (coded as 0).

### Model training and generalization

Of three models, 10-fold cross-validation was used to determine best appropriate data set to further better accuracy on predictions. Firstly, we split the all data into 10 number of folds. Each model has been trained and tested 10 times. For every iteration we selected 1-fold as test data and rest all as training data and ensured that different sets of data were used for model training and testing each time. Finally, we summed up the score for every fold and took the average to get the overall score.

### Evaluation of model performance

The model’s performance was evaluated respectively to compare the sensitivity, specificity, accuracy and area under curve (AUC) of receiver operating characteristic (ROC) of different models for severe COVID-19. The cut-off value was determined by the point on ROC curve where the Youden index was highest.

### Importance of test types

In order to quantify the importance of clinical, laboratory, and C-CT features on the predicted outcomes, the best performance model was used to provide relative feature importance by computing the contribution of each input feature toward the predictive outcome.

### Statistical analysis

Categorical and continuous variables were expressed as *n* (%) and median (interquartile range, IQR), respectively. The Mann Whitney U test, *χ*^2^ test, or Fisher’s exact test were used to compare differences between severe and non-severe cases, as appropriately. The LR, SVM, and RF models were all performed, and AUC were calculated by using python 3.8.3 (Python Software Foundation, Delaware, USA) and anaconda 4.10.1 (Red Hat, Raleigh, North Carolina, USA). Main python module was shown in Additional file 1.

## Results

### Cohort characteristics

A total of 287 patients enrolled in our study. The median age was 60.0 years (IQR: 49.0–68.0 years) with 63.0 years (IQR: 55.5–70.0 years) for severe cases and 57.0 years (IQR: 46.7–67.0 years) for non-severe cases. A higher proportion (56.4%) was male, and the proportion of severe and non-severe cases were 36.6% and 63.4%, respectively. The most common symptoms reported were cough (61.3%) and fever (30.7%), 23 features of COVID-19 patients in the cohort are demonstrated in Table 1.

**Table 1:** Twenty-three features of COVID-19 patients in the cohort

Variables	All cases	Non-severe cases	Severe cases	*P*
	(*n* = 287)	(*n* = 182)	(*n* = 105)	
Fever	88 (30.7)	57 (31.3)	31 (29.5)	0.751
MT	16 (5.6)	11 (6.0)	5 (4.8)	0.648
HR, beats/min	88 (80–99)	87 (80–98)	88 (84–99)	0.122
SBP, mmHg	127 (117–138)	125 (115–136)	131 (121–138)	0.008
Laboratory findings				
NLR	3.28 (2.17–5.77)	2.71 (1.70–4.15)	5.73 (3.17–9.97)	< 0.001
HB	127.0 (117.0–136.0)	128.0 (119.0–136.0)	123 (114.0–136.0)	0.109
RDW-CV	12.4 (11.9–12.9)	12.3 (11.9–12.8)	12.5 (11.9–13.0)	0.032
LDH	258.0 (200.0–346.5)	231.0 (184.7–276.5)	343.0 (261.0–452.0)	< 0.001
IBIL	8.4 (6.1–11.6)	8.9 (6.6–11.8)	7.8 (5.6–10.80)	0.048
D-Dimer	0.7 (0.4–1.5)	0.5 (0.3–0.9)	1.1 (0.6–1.5)	< 0.001
PT	11.3 (10.7–12.0)	11.2 (10.6–11.7)	11.7 (10.8–12.6)	< 0.001
Ca^2+^	2.10 (1.99–2.17)	2.09 (22.0–52.25)	2.01 (1.93–2.10)	< 0.001
ESR	43.0 ( (26.0–58.0)	40.5 (18.0–27.0)	50.3 (35.0–65.5)	< 0.001
AFU	23.0 (19.0–27.0)	23.0 (18.0–27.0)	23.0 (20.0–27.0)	0.508
RBP	26.4 (19.0–40.4)	26.4 (20.6–40.5)	26.4 (17.1–40.0)	0.433
IL-6	8.3 (6.5–11.7)	8.2 (6.5–10.2)	9.2 (7.2–12.9)	0.001
Hs-cTn	3.6 (1.5–8.3)	2.9 (1.1–5.8)	6.2 (2.8–12.4)	< 0.001
AMS	62.0 (49.0–79.5)	62.0 (49.3–74.0)	68.0 (49.0–97.0)	0.078
CysC	0.85 (0.75–1.03)	0.83 (0.73–0.94)	0.95 (0.78–1.16)	< 0.001
IMG	0.01 (0.01–0.04)	0.01 (0.00–0.03)	0.03 (0.01–0.11)	< 0.001
ALP	76.0 (59.5–94.0)	76.0 (59.3–95.8)	75.0 (60.0–93.0)	0.806
MB	42.4 (30.7–64.5)	39.1 (28.0–53.0)	50.1 (38.8–87.3)	< 0.001
C-CT^a^				< 0.001
0	63 (22.0%)	63 (34.6%)	0 (0%)	
1	29 (10.1%)	23 (12.6%)	6 (5.7%)	
2	195 (67.9%)	96 (52.7%)	99 (94.3%)	

Data were presented median (IQR) or *n* (%). *P* values were estimated by comparing variables between severe and non-severe cases

^a^“0” denotes” no lung lesions”, “1” denotes “unilateral lung lesions”, and “2” denotes “bilateral lung lesions”

*MT* Malignant tumor, *HR* Heart rate, *SBP* Systolic blood pressure, *NLR* Neutrophil-to-lymphocyte ratio, *HB* Hemoglobin concentration, *RDW-CV* Red cell volume distribution width, *LDH* Lactate dehydrogenase, *IBIL* Indirect bilirubin, *PT* Prothrombin time, *ESR* Erythrocyte sedimentation rate, *AFU* α-fucosidase, *RBP* Retinol-Binding protein, *IL-6* Interleukin-6, *Hs-cTn* Hypersensitive troponin, *AMS* Amylase, *CysC* cystatin C, *IMG* Immature granulocyte, *ALP* Alkaline phosphatas, *MB* Myoglobin, *C-CT* Chest computed tomography

### Predictive performance

**Fig. 3:**
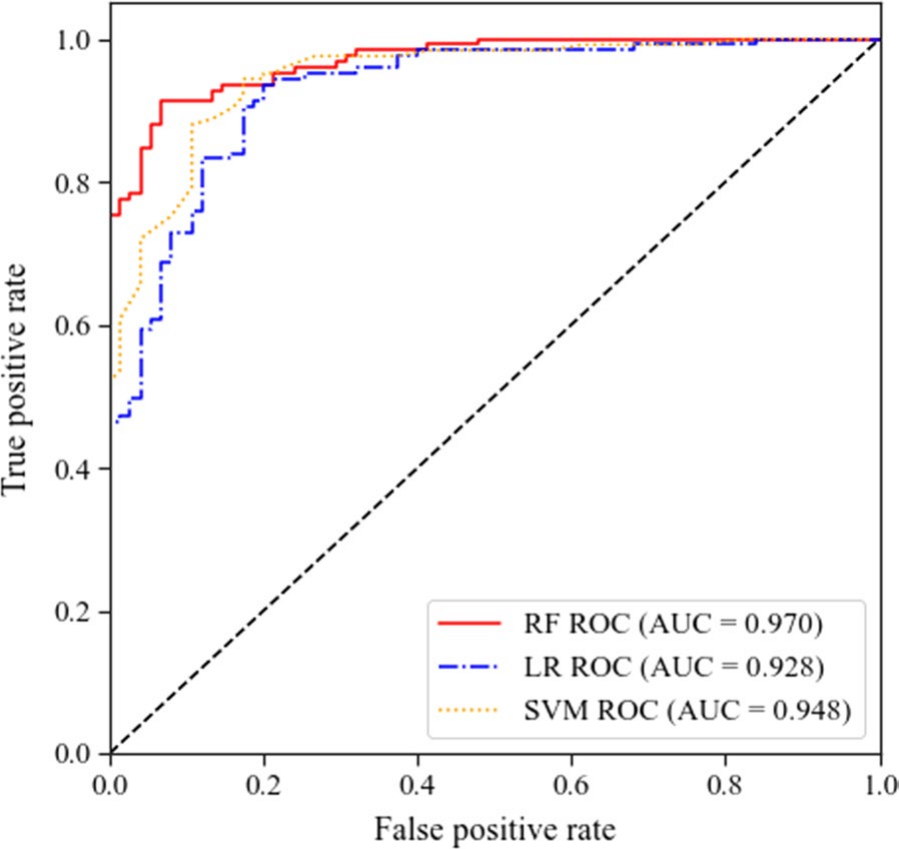
ROC curves of RF, SVM, and LR. *ROC* receiver operating characteristic, *AUC* area under curve, *RF* random forest, *SVM* support vector machine, *LR* logistic regression

We further analyzed the predictive performance of RF, SVM and LR with AUC, sensitivity, specificity, and accuracy (Table 2, Fig. 3), in terms of predictive performance among the three models, we observed that the overall better performance by AUC of 0.970 were RF for predicting COVID-19 severity at admission compared to SVM with AUC of 0.948 and LR with AUC of 0.928. Moreover, RF also achieved a favorable sensitivity of 96.7%, specificity of 69.5%, and accuracy of 84.5%. SVM had sensitivity of 93.9%, specificity of 79.0%, and accuracy of 88.5%. LR model had sensitivity of 92.3%, specificity of 72.3%, and accuracy of 85.2%.

**Table 2:** Predictive performance for COVID-19 severity

Model	AUC	Sensitivity (%)	Specificity (%)	Accuracy (%)	Cut off	YI
RF	0.970	96.7	69.5	84.5	0.62	0.662
SVM	0.948	93.9	79.0	88.5	0.795	0.729
LR	0.928	92.3	72.3	85.2	0.57	0.646

*AUC* area under curve, *YI* youden index, *RF* random Forest, *SVM* support vector machine, *LR* logistic regression

### Feature importance

**Fig. 4:**
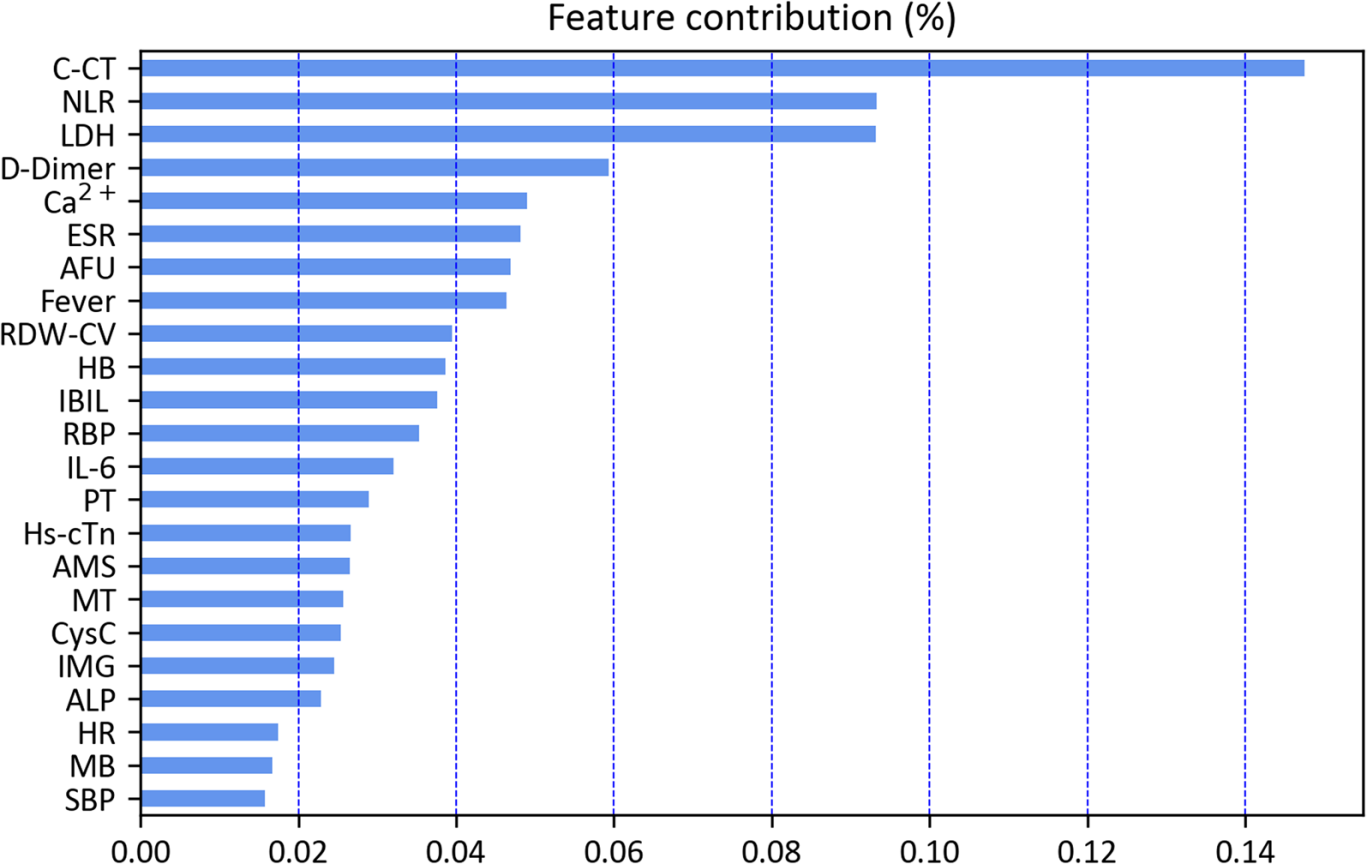
Feature importance: The top 23 features ranked by relative importance to COVID-19 severity by RF. *C-CT* chest computed tomography, *MT* malignant tumor, *HR* heart rate, *SBP* systolic blood pressure, *NLR* neutrophil-to-lymphocyte ratio, *HB* hemoglobin concentration, *RDW-CV* red cell volume distribution width, *LDH* lactate dehydrogenase, *IBIL* indirect bilirubin, *PT* prothrombin time, *ESR* erythrocyte sedimentation rate, *AFU* α-fucosidase, *RBP* retinol-binding protein, *IL-6* interleukin-6, *Hs-cTn* hypersensitive troponin, *AMS* amylase, *CysC* cystatin C, *IMG* immature granulocyte, *ALP* alkaline phosphatas, *MB* myoglobin

We further analyzed the top 23 features ranked by relative importance to COVID-19 severity by using RF with best performance. C-CT had highest importance to COVID-19 severity, and the following were NLR, LDH count, and D-Dimer, respectively (Fig. 4).

## Discussion

Previous studies observed that RF, LR, SVM, gradient boosted decision tree, and neural network to predict COVID-19 [23–25]. Schwab et al. demonstrated that RF had highest predictive performance for predicting hospital admissions for COVID-19 patients, and critical care admissions for COVID-19 cases in terms of AUC compared to LR, SVM, neural network, and gradient boosting [25]. In the present study, we found that RF could predict COVID-19 severity with best performance compared to SVM and LR. The results highlight the possibility that high-performance of RF are able to predict disease severity, the findings can provide reference to monitor parameters effectively and further optimize medical resource.

Chest CT is a widely used imaging test, which can be useful for initial triage of suspected COVID-19 patients. Previous studies demonstrated that chest CT or X-rays findings were associated with COVID-19 severity based on machine learning [26–30], our data observed that chest CT findings with highest importance scores was for diagnosis of severe COVID-19 on admission by RF model, which can help clinicians a fast diagnosis and severity assessment of COVID-19 even before molecular test results are available or laboratory testing is limited. Wang et al. reported that COVID-19 clinical severity could be assessed based on the proposed chest lung-lesion automated deep-learning model [26], given deep learning was likely to be over-fitting for small sample, therefore, which was not used in the study. Following the C-CT ranked feature importance scores were NLR, LDH, D-Dimer, Ca^2+^, ESR, and AFU, respectively, were confirmed in previous studies [30–32]. Ponti et al. reported that NLR was closely in relationship with coagulation cascades in disseminated intravascular coagulation and acute respiratory distress syndrome in severe COVID-19 patients [33]. LDH and D-dimer are consistently reported among hospitalized severe COVID-19 patients [7], while gradual development during the disease course is particularly related to disease worsening in adults and children [34, 35]. However, compared with previous researches, AFU and HB are rarely mentioned in COVID-19 severity. AFU is essential in viral and bacterial infections including the cell-cell adhesion processes that mediate inflammation [36, 37]. Studies illustrated that AFU may be potentially upregulated by chemokines in the later stages of inflammation, such as COVID-19 [37, 38]. We also found that AFU is a significant lab indicator to distinguish severe illness, which may be a possible therapeutic target in the future. In other studies, hemoglobin and red blood cell count were found lower in COVID-19 patients, however, with no significant difference between survivors and non-survivors [39]. COVID-19 patients might suffer from hyper-metabolic states, such as septic shock and multiple organ dysfunction syndromes, which needs an increasing capability of hemoglobin to support the peripheral tissue demands for oxygen [40]. The ability to meet the oxygen demands of the peripheral tissues caused by the COVID-19 infection becomes the leading role to decide the prognosis, according to recent evidence [41].

Our study has several limitations. Firstly, the small sample size from a single study site is a major limitation of the present study, which may undermine reliability of our analysis. Secondly, the data was retrospective collected, some data were incomplete or missing (such as smoking and drinking, and educational level), which were not further analyzed in the study. Further large sample, multi-center studies should be conducted to confirm our results.

## Conclusions

Our study indicates that RF may be a prioritization tool in predicting COVID-19 severity with favorable overall performance at admission. The prediction model can potentially assist clinicians to facilitate severe COVID-19 patients’ identification promptly and apply treatment timely, further optimize potentially healthcare resources.

## Supplementary Information

**Additional file 1.** Main python module.

## Data Availability

The datasets generated and/or analyzed during the present study are not publicly available due to sensitivity of the patients’ personal information but are available from the corresponding author on reasonable request.

## Abbreviations

AFU: α-Fucosidase
ALB: Albumin
ALP: ALkaline phosphatas
AMS: Amylas
ARDS: Acute respiratory distress syndrome
AUC: Area under curve
CHE: Cholinesterase
CK: Creatine kinase
COVID-19: Coronavirus disease 2019
CT: Computed tomography
CysC: Cystatin C
DIC: Disseminated intravascular coagulation
ESR: Erythrocyte sedimentation rate
HB: Hemoglobin concentration
HCT: Hematocrit
HR: Heart rate
hs-cTn: Hypersensitive troponin
IBIL: Indirect bilirubin
IL-6: Interleukin-6
IMG: Immature granulocyte
LASSO: Least Absolute Shrinkage and Selectionator Operator
LDH: Lactate dehydrogenase
LR: Logistic regression
MB: Myoglobin
MT: Malignant tumor
NLR: Neutrophil-to-lymphocyte ratio
PT: Prothrombin time
RBP: Retinol-binding protein
RBC: Red blood cell
RDW-CV: Red cell volume distribution width
RF: Random forest
ROC: Receiver operating characteristic
γ-GT: γ-Glutamyl transpeptidase
SBP: Systolic blood pressure
SVM: Support vector machine
WBC: White blood cell
YI: Youden index

## References

[1] Zhu J, Yan W, Zhu L, Liu J (2021). COVID-19 pandemic in BRICS countries and its association with socio-economic and demographic characteristics, health vulnerability, resources, and policy response. Infect Dis Poverty.

[2] Ma Y, Mishra SR, Han XK, Zhu DS (2021). The relationship between time to a high COVID-19 response level and timing of peak daily incidence: an analysis of governments' Stringency Index from 148 countries. Infect Dis Poverty.

[3] Geng MJ, Wang LP, Ren X, Yu JX, Chang ZR, Zheng CJ (2021). Risk factors for developing severe COVID-19 in China: an analysis of disease surveillance data. Infect Dis Poverty.

[4] Zhou F, Yu T, Du R, Fan G, Liu Y, Liu Z (2020). Clinical course and risk factors for mortality of adult inpatients with COVID-19 in Wuhan, China: a retrospective cohort study. Lancet.

[5] Hu B, Guo H, Zhou P, Shi ZL (2021). Characteristics of SARS-CoV-2 and COVID-19. Nat Rev Microbiol.

[6] Li X, Xu S, Yu M, Wang K, Tao Y, Zhou Y (2020). Risk factors for severity and mortality in adult COVID-19 inpatients in Wuhan. J Allergy Clin Immunol.

[7] Zheng Z, Peng F, Xu B, Zhao J, Liu H, Peng J (2020). Risk factors of critical & mortal COVID-19 cases: a systematic literature review and meta-analysis. J Infect.

[8] Xiong Y, Ma Y, Tian Y, Zhang C, Yang W, Liu B (2021). A longitudinal cohort study using a modified child-pugh score to escalate respiratory support in COVID-19 patients—Hubei Province, China, 2020. China CDC Wkly.

[9] Bai XY, Xin TY, Yan M, Wei Y, Bin L, Guo RL (2021). Factors defining the development of severe illness in patients with COVID-19: a retrospective study. Biomed Environ Sci.

[10] Xu PP, Tian RH, Luo S, Zu ZY, Fan B, Wang XM (2020). Risk factors for adverse clinical outcomes with COVID-19 in China: a multicenter, retrospective, observational study. Theranostics.

[11] Silva K, Lee WK, Forbes A, Demmer RT, Barton C, Enticott J (2020). Use and performance of machine learning models for type 2 diabetes prediction in community settings: a systematic review and meta-analysis. Int J Med Inform.

[12] Krittanawong C, Virk HUH, Bangalore S, Wang Z, Johnson KW, Pinotti R (2020). Machine learning prediction in cardiovascular diseases: a meta-analysis. Sci Rep.

[13] Lu W, Fu D, Kong X, Huang Z, Hwang M, Zhu Y (2020). FOLFOX treatment response prediction in metastatic or recurrent colorectal cancer patients via machine learning algorithms. Cancer Med.

[14] Castaldo R, Cavaliere C, Soricelli A, Salvatore M, Pecchia L, Franzese M (2021). Radiomic and genomic machine learning method performance for prostate cancer diagnosis: systematic literature review. J Med Internet Res.

[15] Fleuren LM, Klausch TLT, Zwager CL, Schoonmade LJ, Guo T, Roggeveen LF (2020). Machine learning for the prediction of sepsis: a systematic review and meta-analysis of diagnostic test accuracy. Intensive Care Med.

[16] Lee Y, Ragguett RM, Mansur RB, Boutilier JJ, Rosenblat JD, Trevizol A (2018). Applications of machine learning algorithms to predict therapeutic outcomes in depression: A meta-analysis and systematic review. J Affect Disord.

[17] Kwon JM, Kim KH, Jeon KH, Lee SE, Lee HY, Cho HJ (2019). Artificial intelligence algorithm for predicting mortality of patients with acute heart failure. PLoS ONE.

[18] Schalekamp S, Huisman M, van Dijk RA, Boomsma MF, Freire Jorge PJ, de Boer WS (2021). Model-based prediction of critical illness in hospitalized patients with COVID-19. Radiology.

[19] Wynants L, Van Calster B, Collins GS, Riley RD, Heinze G, Schuit E (2020). Prediction models for diagnosis and prognosis of covid-19: systematic review and critical appraisal. BMJ.

[20] Wu G, Yang P, Xie Y, Woodruff HC, Rao X, Guiot J (2020). Development of a clinical decision support system for severity risk prediction and triage of COVID-19 patients at hospital admission: an international multicentre study. Eur Respir J.

[21] Li WT, Ma J, Shende N, Castaneda G, Chakladar J, Tsai JC (2020). Using machine learning of clinical data to diagnose COVID-19: a systematic review and meta-analysis. BMC Med Inform Decis Mak.

[22] Diagnosis and Treatment Protocol for Novel Coronavirus Pneumonia (Trial Version 7). Chin Med J (Engl). 2020; 133:1087–1095. 10.1097/CM9.0000000000000819 PMC7213636 32358325

[23] Yue H, Yu Q, Liu C, Huang Y, Jiang Z, Shao C (2020). Machine learning-based CT radiomics method for predicting hospital stay in patients with pneumonia associated with SARS-CoV-2 infection: a multicenter study. Ann Transl Med.

[24] Gao Y, Cai GY, Fang W, Li HY, Wang SY, Chen L (2020). Machine learning based early warning system enables accurate mortality risk prediction for COVID-19. Nat Commun.

[25] Schwab P, DuMont SA, Dietz B, Bauer S (2020). Clinical predictive models for COVID-19: systematic study. J Med Internet Res.

[26] Wang G, Liu X, Shen J, Wang C, Li Z, Ye L (2021). A deep-learning pipeline for the diagnosis and discrimination of viral, non-viral and COVID-19 pneumonia from chest X-ray images. Nat Biomed Eng.

[27] Li L, Qin L, Xu Z, Yin Y, Wang X, Kong B (2020). Using artificial intelligence to detect COVID-19 and community-acquired pneumonia based on pulmonary CT: evaluation of the diagnostic accuracy. Radiology.

[28] Bouchareb Y, Moradi Khaniabadi P, Al Kindi F, Al Dhuhli H, Shiri I, Zaidi H (2021). Artificial intelligence-driven assessment of radiological images for COVID-19. Comput Biol Med.

[29] Reeves RA, Pomeranz C, Gomella AA, Gulati A, Metra B, Hage A (2021). Performance of a severity score on admission chest radiography in predicting clinical outcomes in hospitalized patients with coronavirus disease (COVID-19). AJR Am J Roentgenol.

[30] Chen Y, Ouyang L, Bao FS, Li Q, Han L, Zhang H (2021). A multimodality machine learning approach to differentiate severe and nonsevere COVID-19: Model development and validation. J Med Internet Res.

[31] Liu J, Liu Y, Xiang P, Pu L, Xiong H, Li C (2020). Neutrophil-to-lymphocyte ratio predicts critical illness patients with 2019 coronavirus disease in the early stage. J Transl Med.

[32] Feng DY, Zhou YQ, Zhou M, Zou XL, Wang YH, Zhang TT (2019). Risk factors for mortality due to ventilator-associated pneumonia in a Chinese hospital: a retrospective study. Med Sci Monit.

[33] Ponti G, Maccaferri M, Ruini C, Tomasi A, Ozben T (2020). Biomarkers associated with COVID-19 disease progression. Crit Rev Clin Lab Sci.

[34] Terpos E, Ntanasis-Stathopoulos I, Elalamy I, Kastritis E, Sergentanis TN, Politou M (2020). Hematological findings and complications of COVID-19. Am J Hematol.

[35] Zhou MY, Xie XL, Peng YG, Wu MJ, Deng XZ, Wu Y (2020). From SARS to COVID-19: what we have learned about children infected with COVID-19. Int J Infect Dis.

[36] Tu Z, Lin YN, Lin CH (2013). Development of fucosyltransferase and fucosidase inhibitors. Chem Soc Rev.

[37] Liu T, Liu R, Zhu L, Zou X, Guan H, Xu Z (2019). Development of a UHPLC-MS method for inhibitor screening against α-l-1,3-fucosidase. Anal Bioanal Chem.

[38] Ali S, Jenkins Y, Kirkley M, Dagkalis A, Manivannan A, Crane IJ (2008). Leukocyte extravasation: an immunoregulatory role for alpha-l-fucosidase?. J Immunol.

[39] Taneri PE, Gómez-Ochoa SA, Llanaj E, Raguindin PF, Rojas LZ, Roa-Díaz ZM (2020). Anemia and iron metabolism in COVID-19: a systematic review and meta-analysis. Eur J Epidemiol.

[40] Lin GL, McGinley JP, Drysdale SB, Pollard AJ (2018). Epidemiology and immune pathogenesis of viral sepsis. Front Immunol.

[41] Gattinoni L, Coppola S, Cressoni M, Busana M, Rossi S, Chiumello D (2020). COVID-19 does not lead to a "typical" acute respiratory distress syndrome. Am J Respir Crit Care Med.

