# Supplementary material for: Comparing different machine learning techniques for predicting COVID-19 severity
Source: Infect Dis Poverty. 2022 Feb 17;11:19. doi: 10.1186/s40249-022-00946-4 (PMC8851750; doi:10.1186/s40249-022-00946-4)
Supplement: Supplementary file 1 — Additional file 1. Main python module. [file 40249_2022_946_MOESM1_ESM.pdf]

Main python module

```
from sklearn.svm import SVC
from sklearn.model_selection import train_test_split
import matplotlib.pyplot as plt
import numpy as np
from time import time
import datetime

from sklearn import tree
from sklearn.model_selection import train_test_split
import pandas as pd
import numpy as np
from sklearn.preprocessing import OrdinalEncoder
from sklearn.preprocessing import KBinsDiscretizer
from sklearn.preprocessing import LabelEncoder, OneHotEncoder
from six import StringIO
from sklearn import tree
from sklearn import preprocessing
import pydotplus
from sklearn.feature_extraction import DictVectorizer
## HD image output
%config InlineBackend.figure_format = 'retina'
%matplotlib inline

import matplotlib
matplotlib.rcParams['axes.unicode_minus']=False
import seaborn as sns
sns.set(font= "Kaiti",style="ticks",font_scale=1.4)
import pandas as pd
pd.set_option("max_colwidth", 200)

import warnings
warnings.filterwarnings("ignore")
from sklearn import tree
from sklearn.model_selection import train_test_split
import pandas as pd
import numpy as np
from sklearn.preprocessing import OrdinalEncoder
from sklearn.preprocessing import KBinsDiscretizer
from sklearn.preprocessing import LabelEncoder, OneHotEncoder
from six import StringIO
```

```
from sklearn import tree
from sklearn import preprocessing
import pydotplus
from sklearn.feature_extraction import DictVectorizer
import numpy as np
import pandas as pd
import datetime

from sklearn.linear_model import LogisticRegression
from sklearn.ensemble import RandomForestClassifier
from sklearn.model_selection import GridSearchCV
from sklearn.model_selection import train_test_split
from sklearn.metrics import confusion_matrix
from sklearn.metrics import precision_recall_curve
from sklearn.metrics import auc
from sklearn.metrics import roc_auc_score
from sklearn.metrics import roc_curve
from sklearn.metrics import recall_score
from sklearn.metrics import classification_report
from sklearn.metrics import accuracy_score
from sklearn.preprocessing import StandardScaler

import warnings
warnings.filterwarnings('ignore')
pd.set_option('display.float_format', lambda x: '%.4f' % x)
from imblearn.over_sampling import SMOTE
import itertools
# Data processing
data_shuju=pd.read_excel('data.xlsx')
data_shuju.set_index('ID', inplace=True)
data_shuju.head()
from sklearn.preprocessing import LabelEncoder
# Outcome variable coding processing
y = data_shuju.iloc[:,-1]
le = LabelEncoder()
le = le.fit(y)
label = le.transform(y)
label
le.classes_
y = label

# Modeling independent variables were extracted
x = data_shuju.iloc[:,0:-1]
x.head()
```

```
#Start the modeling
# Random forest modeling
# Random Forest
#Adjust n_estimators
param_test={'n_estimators':range(10,101,10)}
gsearch = GridSearchCV(estimator=RandomForestClassifier(),
          param_grid=param_test, cv=10, scoring='accuracy')

gsearch.fit(x,y)
print(gsearch.best_params_,gsearch.best_score_)

#Adjust max_depth
param_test={'max_depth':range(2,12,2)}   # Setting parameter range
gsearch = GridSearchCV(estimator=RandomForestClassifier(n_estimators=80),
          param_grid=param_test, cv=10, scoring='accuracy')

gsearch.fit(x,y)
print(gsearch.best_params_,gsearch.best_score_)
#Adjust min_samples_leaf
param_test={'min_samples_leaf':range(2,8,1)}   # Setting parameter range
gsearch = GridSearchCV(estimator=RandomForestClassifier(n_estimators=80,max_depth=3),
          param_grid=param_test, cv=10, scoring='accuracy')

gsearch.fit(x,y)
print(gsearch.best_params_,gsearch.best_score_)


# Evaluation index of random forest calculation model
# Points judged as 1 / all points judged as 1, sensitivity
(y[y == gsearch.predict(x)] == 1).sum()/(y == 1).sum()
# Points judged as 0 / all points judged as 0, specificity
(y[y == gsearch.predict(x)] == 0).sum()/(y == 0).sum()
# Judged correctly and truly 1 / all parts judged as 1, accuracy
(y[y == gsearch.predict(x)] == 1).sum()/(gsearch.predict(x) == 1).sum()

# The ROC curve was drawn by random forest and the cutoff value was calculated
def Find_Optimal_Cutoff(TPR, FPR, threshold):
    y = TPR - FPR
    Youden_index = np.argmax(y)  # Only the first occurrence is returned.
    optimal_threshold = threshold[Youden_index]
    point = [FPR[Youden_index], TPR[Youden_index]]
    return optimal_threshold, point
```

```
plt.figure()

plt.figure(figsize=(5,5))
plt.plot(fpr, tpr, color='red',
        label='ROC curve (area = %0.2f)' % roc_auc)

plt.plot([0, 1], [0, 1], color='black',  linestyle='--')
plt.xlim([0.0, 1.0])
plt.ylim([0.0, 1.05])

optimal_th, optimal_point = Find_Optimal_Cutoff(TPR=tpr, FPR=fpr, threshold=thresholds)
plt.plot(optimal_point[0], optimal_point[1], marker='o', color='r')
plt.text(optimal_point[0], optimal_point[1], f'Threshold:{optimal_th:.2f}')

y_pred1_prob = gsearch.predict_proba(x)[:, 1] # The default threshold is 0.5

fpr, tpr, thresholds = roc_curve(y,y_pred1_prob)
roc_auc = auc(fpr,tpr)
plt.xlabel('False Positive Rate')
plt.ylabel('True Positive Rate')
#plt.title('Receiver operating characteristic example')
plt.legend(loc="lower right")

# Logistic regression modeling
param_grid = {'C': [0.01,0.1, 1, 10, 100],
                         'penalty': [ 'l1', 'l2']}

grid_search   =   GridSearchCV(LogisticRegression(),     param_grid,   cv=10)
LogisticRegression，  param_grid ， cv as 10-fold cross-validation method was used
grid_search.fit(x, y) # Using training set learning algorithm
results = pd.DataFrame(grid_search.cv_results_)
best = np.argmax(results.mean_test_score.values)
print("Best parameters: {}".format(grid_search.best_params_))
print("Best cross-validation score: {:.5f}".format(grid_search.best_score_))

# Evaluation index of logistic regression calculation model
# Points judged as 1 / all points judged as 1, sensitivity
(y[y == grid_search.predict(x)] == 1).sum()/(y == 1).sum()
# Points judged as 0 / all points judged as 0, specificity
(y[y == grid_search.predict(x)] == 0).sum()/(y == 0).sum()
# Judged correctly and truly 1 / all parts judged as 1, accuracy
(y[y == grid_search.predict(x)] == 1).sum()/(grid_search.predict(x) == 1).sum()

# The ROC curve was drawn by logistic regression and the cutoff value was calculated
```

```
def Find_Optimal_Cutoff(TPR, FPR, threshold):
    y = TPR - FPR
    Youden_index = np.argmax(y)  # Only the first occurrence is returned.
    optimal_threshold = threshold[Youden_index]
    point = [FPR[Youden_index], TPR[Youden_index]]
    return optimal_threshold, point


y_pred1_prob = grid_search.predict_proba(x)[:, 1]  The default threshold is 0.5

fpr, tpr, thresholds = roc_curve(y,y_pred1_prob)
roc_auc = auc(fpr,tpr)

plt.figure()

plt.figure(figsize=(5,5))
plt.plot(fpr, tpr, color='red',
         label='ROC curve (area = %0.2f)' % roc_auc)

plt.plot([0, 1], [0, 1], color='black',  linestyle='--')
plt.xlim([0.0, 1.0])
plt.ylim([0.0, 1.05])

optimal_th, optimal_point = Find_Optimal_Cutoff(TPR=tpr, FPR=fpr, threshold=thresholds)
plt.plot(optimal_point[0], optimal_point[1], marker='o', color='r')
plt.text(optimal_point[0], optimal_point[1], f'Threshold:{optimal_th:.2f}')

plt.xlabel('False Positive Rate')
plt.ylabel('True Positive Rate')
plt.title('Receiver operating characteristic example')
plt.legend(loc="lower right")

# Support vector machine modeling
Kernel = ["linear","poly","rbf","sigmoid"]
for kernel in Kernel:
    time0 = time()
    clf = SVC(kernel = kernel
            ,gamma ="auto"
            ,degree = 1
            ,cache_size = 5000
            ).fit(xtrain,ytrain)
    print("The accuracy under kernel %s is %f" % (kernel,clf.score(xtest,ytest)))
    print(datetime.datetime.fromtimestamp(time()-time0).strftime("%M:%S:%f"))
score =[]
```

```
C_range = np.linspace(0.01,3,50)
for i in C_range:
    clf = SVC(kernel = "linear",C =i ,cache_size= 9000).fit(xtrain,ytrain)
    score.append(clf.score(xtest,ytest))

print(max(score),C_range[score.index(max(score))])
plt.plot(C_range,score)
plt.show()

clf_proba = SVC(kernel = "linear",C = 6.1263265306122445,probability = True).fit(x,y)
# Evaluation index of support vector machine calculation model
# Points judged as 1 / all points judged as 1, sensitivity
(y[y == clf_proba.predict(x)] == 1).sum()/(y == 1).sum()
# Points judged as 0 / all points judged as 0, specificity
(y[y == clf_proba.predict(x)] == 0).sum()/(y == 0).sum()
# Judged correctly and truly 1 / all parts judged as 1, accuracy
(y[y == clf_proba.predict(x)] == 1).sum()/(clf_proba.predict(x) == 1).sum()
# The ROC curve was drawn by support vector machine and the cutoff value was calculated
probrange =
np.linspace(clf_proba.predict_proba(x)[:,1].min(),clf_proba.predict_proba(x)[:,1].max(),num =
50,endpoint = False)

from sklearn.metrics import confusion_matrix as CM, recall_score as R
import matplotlib.pyplot as plot
# AUC was calculated
from sklearn.metrics import roc_auc_score as AUC

area = AUC(y,clf_proba.decision_function(x))
area
recall=[]
FPR = []

for i in probrange:
    y_predict = []
    for j in range(x.shape[0]):
        if clf_proba.predict_proba(x)[j,1]>i:
            y_predict.append(1)
        else:
            y_predict.append(0)
    cm = CM(y,y_predict,labels = [1,0])
    recall.append(cm[0,0]/cm[0,:].sum())
    FPR.append(cm[1,0]/cm[1,:].sum())

recall.sort()
```

```
FPR.sort()

plt.plot(FPR,recall, color = "orange",linestyle=':',
        label = 'SVM ROC curve (area = %0.3f)' % area)
plt.plot([0, 1], [0, 1], color='black',  linestyle='--')
plt.xlim([0.0, 1.0])
plt.ylim([0.0, 1.05])
plt.xlabel('False Positive Rate')
plt.ylabel('True Positive Rate')
#plt.title('Receiver operating characteristic example')
plt.legend(loc="lower right")
# Calculate the cutoff value
recall=np.array(recall)
FPR=np.array(FPR)
maxindex = (recall - FPR).tolist().index(max(recall - FPR))
print(thresholds[maxindex])
```
